# Applications and Challenges of Telemedicine: Privacy-Preservation as a Case Study

**DOI:** 10.34172/aim.2023.96

**Published:** 2023-11-01

**Authors:** Maryam Ansarian, Zahra Baharlouei

**Affiliations:** ^1^Medical Image and Signal Processing Research Center, Isfahan University of Medical Sciences, Isfahan, Iran

**Keywords:** Digital health, Privacy-Preserving, Telehealth, Telemedicine

## Abstract

Today, technology has an important impact on the development of medical services, especially during the outbreak of COVID-19. Telemedicine, known by terms such as telehealth and digital health, refers to the utilization of technology to provide health care services at a distance that leads to improved monitoring, detecting and treatment of disease, and provision of individual care. It has been considered in various fields such as radiology, cardiology, pulmonology, psychiatry, emergency care and surgery. The most important advantages of using telemedicine are saving time for the doctor and the patient, reducing the cost of multiple visits to the doctor, reducing the spread of contagious diseases and caring for patients who cannot see a doctor, such as the elderly. In this paper, we review the research in the field of applying telemedicine, as well as its advantages and disadvantages. Next, we discuss the challenges in the field of using telemedicine which are privacy preserving, data security, cost of infrastructures, lack of physical examination and responsibility for patients’ compensation. One of the most important challenges is privacy preserving of patients’ information during transmission and process. We categorize and compare the various methods that have been proposed to protect peoples’ privacy.

## Introduction

 In the field of remote care, various terms are used, which are briefly explained in the following. The terms telehealth and telemedicine are often used interchangeably. Telemedicine refers specifically to clinical services while telehealth refers to the use of telecommunication in health care delivery and information according to the health resources and services administration. Telehealth and telemedicine cover similar services, including remote monitoring and consultation of patients, medical education, wireless health applications, and transmission of medical reports.^1,2^ In other words, telemedicine, telehealth, or eHealth all mean using technology to provide healthcare services to patients remotely in terms of diagnosis, treatment, and prevention of diseases or injuries.^3^ The term digital health refers to the use of media, platforms, and applications that are connected to the internet to improve medical diagnosis and disease treatment for patients.^4^ The term M-Health emphasizes the use of mobile phones in healthcare services.^5^

 Research in the field of telemedicine is extensive. Some have discussed the use of telemedicine for different issues such as COVID-19,^6^ Diabetes,^7^ Ophthalmology,^8^ Mental health,^9^ Heart,^10,11^ Pregnancy,^12^ Nursing,^13^ Respiratory,^14^ Cancer,^15^ Dermatology,^16^ Dentistry ^17^ and Insomnia.^18^ Several papers have explained the benefits of telemedicine; for example, quick access to medical care especially in remote areas,^2^ reducing the cost of multiple visits to the doctor,^19,20^ and prolonged hospitalizations.^21,22^ Some research has been focused on the challenges and limitations in telemedicine such as data security and privacy,^23,24^ the cost of infrastructure,^2,3^ lack of available equipment such as high-speed internet,^25,26^ data accuracy,^4,6^ and misdiagnosis.^3,23^ Since one of the most important problems in telemedicine is preserving the privacy of patients’ information, several papers have investigated and provided solutions for this problem such as Blockchain based,^27-30^ Graph based,^31-34^ Watermarking algorithms,^35-38^ Identity based,^39,40^ Homomorphic,^41^ and Attribute based signature.^42^

 Several review papers in this area are mentioned in the following. In one study,^20^ the authors investigated the current status of telemedicine and identified what is still needed to be implemented in telemedicine regarding ethical and legal standards. They concluded that there is a gap between current service providers and most patient interactions with the use of data. In another study,^3^ guidelines on telehealth and telemedicine in South East Asian countries were compared. The results of this study showed that there should be a comprehensive and universal telemedicine guideline for each country to ensure uniformity of telemedicine services and patient safety. One study^43^ discussed the cyber-physical system perspective of digital health devices and proposed a health management informatics framework for the devices providing health data. The use of telemedicine in different countries during the COVID-19 pandemic and how to increase the adoption of remote health technologies in the future were explored in another study.^44^ A systematic review^45^ was conducted on the challenges and barriers of using telemedicine. The results demonstrated that although telemedicine became widespread during the COVID-19 pandemic, there are still important challenges that should be overcome to optimize its utilization. Another review^46^ studied many papers to evaluate the satisfaction of patients with telemedicine during the COVID-19 pandemic. It reported a high level of satisfaction with telehealth in the studies despite different challenges that must be eliminated by patients’ feedback. In another study,^47^ the benefits and drawbacks of using telemedicine in the emergency department (ED) were explored. It was concluded that telemedicine has a high potential to improve patient care in ED; however, further studies are needed to confirm its feasibility. Several review papers have investigated the applications of telemedicine in specific diseases like rheumatology,^48^ cardiology,^49^ orthopedic surgery,^50^ diabetes,^51^ and neurology.^52^ The result of all of these studies show the feasibility and effectiveness of telemedicine and the need for more research to enhance its long-term utilization.

 In this review paper, first, the applications of telemedicine are presented. Afterwards, the advantages and disadvantages of applying telemedicine are explained. Finally, the challenges and their solutions are discussed. We focus on the privacy-preserving problem, as one of the most important challenges in this field. It should be noted that if people are not sure that their privacy will be protected, they will not accept the telemedicine technology. Privacy includes basic information about people, their diseases and records, and other private things that people do not want to be shared with others. Therefore, this issue plays a fundamental role in the implementation of telemedicine, and appropriate algorithms must be used to protect people’s privacy. Several algorithms have been proposed in the literature which we will classify and compare at the end.

## Telemedicine Application

 Recently, telemedicine has become feasible and well-accepted in various areas, as a result of the development of digital technologies.^6^ In the era of COVID-19, mandatory remote connections have made telemedicine the best way for treatment.^53,54^ Tele-ophthalmology is an effective method for people with acute ocular conditions.^8,55^ It is mostly used for observing retinal diseases like diabetic retinopathy, macular degeneration and glaucoma.^56^ Tele-psychiatry proved to be better than in-person visits for patients who had difficulty building trust with new physicians.^57^ Tele-psychiatry also needs more primary care than referral care and creates more opportunities for physicians to learn about mental health diagnosis and treatment. The referral approach is more effective for patients with higher complexity or a desire for specific psychotherapy.^9,58^ In patients suffering from chronic respiratory diseases, the possibility of monitoring different physiological parameters of patients during their daily routine activities using digital devices is one of the advantages of remote treatment.^14^ Information is stored on a smartphone and available to the doctor. Based on this information, the doctors will be able to adjust the drugs and doses according to the needs of each patient.^24,59^ Today, despite the increasing prevalence of all types of cancer worldwide, there are inadequate treatments, physicians and surgeons, especially in rural areas.^60-62^ Therefore, using distance care technology for diagnosis, treatment, and supportive care increases access to cancer care and improves physician education.^15,63^

 Tele-dermatology is essential for underserved communities to access healthcare. Due to the spread of the COVID-19 infection, tele-dermatology will increase access to care and reduce disparities among many populations through convenience, efficiency, and affordability.^19,36,64,65^ Remote dentistry can be a supplement to in-person dental care methods, especially in children, and ultimately lead to better management of the patients.^66,67^ This technology can significantly help in places where healthcare facilities are limited and ensure safety during a pandemic while providing dental care to pediatric patients.^17,68,69^ More research is needed on the safe and effective use of tele-dentistry in pediatric dentistry.^70^ In people with insomnia, deep learning methods can evaluate the quality of sleep based on the information recorded by the person during his waking hours on smartphones.^18,71^ These predictive methods can be an effective way for sleep research and improve eHealth solutions for sleep.^72^

 Globally, the number of elderly people is increasing. Today, nursing homes struggle with staffing shortages and access to specialty care, while simultaneously facing increasing pressure to reduce unavoidable hospital and ED admissions.^13,73,74^ The use of remote care helps to provide specialized facilities and home care for the elderly.^75^ Most of all, people with cardiovascular diseases and chronic respiratory disorders need such care.^76-78^ In remote care, vital sign recorders and video communication devices are installed in the patient’s home and patients’ information such as heart rate, blood pressure, breathing, and the image of the patient are sent to the nurse.^79^ In Table 1, we categorize the papers in the literature based on different diseases and methods or devices that are used in telemedicine.

**Table 1:** Telemedicine in Different Diseases

**Types of Disease**	**Device / Method**
COVID-19	Mobile phone, video conferencing, machine learning
Diabetes	Wearable sensors, Internet of medical things
Ophthalmology	Video conferencing
Mental health	Mobile phone, video conferencing
Heart	Mobile phone
Pregnancy	Mobile application
Cancer	Video conferencing
Dermatology	Electronic device, Internet connection
Insomnia	Smart phone, deep learning method
Nursing	Video conferencing
Respiratory	Digital devices like smart phone
Dentistry	Mobile phone, computer

## Benefits and Barriers of Telemedicine

 Telemedicine provides many advantages such as quick access to doctors at a lower cost, elimination of doctor and patient distances especially in remote and inaccessible areas, having the support of medical specialists, nursing, or psychological team at any time, using the patient information bank to check the process of disease improvement, ease of exchange of laboratory results and radiology images, reducing patient travel and stress which will lead to improved patient satisfaction, reducing the spread of contagious diseases and taking care of patients who cannot see a doctor, such as the elderly, pregnant people and people with underlying diseases.^2,19,20,67,80-82^

 Telemedicine has also the potential to eliminate healthcare problems such as medical misuse, unnecessary visits, and long-term hospitalizations.^21,22,83,84^

 Some disadvantages of telehealth include restrictions on complete physical examinations, the possibility of technical problems like equipment failure during examination and treatment and loss of privacy during storage and use of patients’ information. Telehealth may negatively impact continuity of care because in virtual interactions, the physician does not have the benefit of thorough history and physical examination to aid in diagnosis and treatment.^3,23^ In many cases, a physical examination is required for an accurate diagnosis, so an in-person visit is necessary, and telehealth should complement the in-person visit. Also, compared to in-person examinations, telemedicine encounters are more vulnerable to privacy and security risks.^85,86^ Another important obstacle in telemedicine is transmission accuracy. Internet bandwidth influences the validity and reliability of data which can lead to decisions and treatment recommendations by doctors based on incorrect patient data. Mainly, in remote areas, there is not enough bandwidth for the correct transmission of information, and the use of this technology in these areas requires the necessary infrastructure.^2^ Another obstacle in the field of telemedicine is limited access to the internet or smartphones, tablets, and computers in some areas. Lack of knowledge about technology, computer illiteracy, and language barriers between the provider and the patient are also possible. In addition, a virtual visit may not be suitable for some patients with specific conditions. Problems such as the high cost of implementation, lack of a legal framework including patient privacy policies, and health professional authentication are also major obstacles to wide acceptance and use of telemedicine.^24-26^

 In addition to the mentioned obstacles, compliance with ethical requirements and considerations such as compilation of comprehensive and global guidelines for recording and using patients’ information, the competence of the treatment staff, the liability of medical malpractice, obtaining informed consent, providing insurance for damages, training providers, user-friendly technology, reliability, availability and the existence of high quality software are essential.^4,84^ We categorize the advantages and disadvantages of telemedicine in Table 2.

**Table 2:** Advantages and Disadvantages of Telemedicine

**Advantages**	**Disadvantages**
Quick access to health facilities	Data security and privacy
Saving time for doctors and patients	High cost of infrastructure
Reducing the cost of multiple visits to the doctor	Lack of available equipment such as high-speed internet
Reducing the spread of disease	Lack of training, lack of skilled labor
Using the patient information bank to check the process of disease improvement	Lack of a comprehensive and complete physical examination
Ease of exchange of laboratory results, radiology images	The possibility of a decrease in the quality of healthcare
Improving the provision of medical services to rural and remote areas	The possibility of technical problems during the examination
Exchange of new medical findings between doctors around the world	Data accuracy and misdiagnosis
Having the support of medical specialists, nursing, or psychological team at any time	Uncertainty of patient eligibility for telecare (may require in-person care)
Reducing stress and prolonged hospitalizations	Absence of specific instructions for the person who is responsible for damages

 One of the main concerns in the field of telemedicine is preserving the privacy of personal information that is stored and transmitted through digital devices.^6^ This information can be the target of cyber-attacks that lead to the disclosure of patients’ private information.^87^ Since this issue plays a fundamental role in the implementation of telemedicine, we focus on this case and review it in more details in the following section.

## Privacy Preserving in Telemedicine

 Privacy refers to the right to access ones information or identity and to participate in data processing decisions, such as disclosure, preservation, and elimination.^88^ Currently, many privacy issues related to telehealth are caused by lack of knowledge among employees about the privacy of information when using medical equipment. Therefore, medical care providers must have adequate training and competence to manage patients remotely. Healthcare organizations must increase their security by updating the devices’ software. Also, there should be a comprehensive guideline for telemedicine for each country in which personal information, data ownership, backups, cyber security, and solutions to overcome the limitations of telemedicine in comparison to in-person examinations should be defined to guarantee the uniformity of telemedicine services and patient privacy.^79^

 Various methods have been considered to preserve privacy in telemedicine. For example, one study^27^ examined the benefits and challenges of adapting the blockchain technology in the field of telemedicine and the key role of this method in ensuring data privacy, the immutability of health records, and traceability to discover patients’ insurance fraud. It was concluded that the blockchain technology can develop telehealth services by providing telehealth care in a decentralized, transparent, traceable, reliable, and safe manner. It was estimated that the adoption of blockchain could save about 100 billion dollars per year in data security costs by 2025.^28^ The wide use of blockchain in telemedicine is still in its early stages, and various challenges should be overcome before extensive adoption of the blockchain technology in telehealth systems.^27^ Connecting blockchain to current healthcare systems is complex and expensive.^28^ Therefore, the cost, lack of knowledge about execution, and lack of standardization are the main challenges that prevent the admission of blockchain in telemedicine.^29^

 There are other methods to increase the privacy of data in telemedicine in the form that the telemedicine network is considered in the form of a graph, which includes the source node, as the place where information is collected and the sink node as a place to store and process information by processing and machine learning algorithms and the paths through which, information is sent from the source node to the sink node.^31^ For example, in one study,^32^ data was collected intensively using the homomorphic function, and the security of the data was guaranteed using encryption. The authors of another study ^33^ proposed a method to find the location of the sink node at the same time with the least energy consumption. In another research,^34^ random data collection methods were used to maintain the location of the sink node, and the data was sent through random paths that cannot be easily identified. In these methods, data is transferred from the source node from one point to another to reach the sink node, and the sink node sends data as the last destination, which is different from other nodes and is easy to track. This route is detected by hackers.

 One study^31^ proposed a method to solve the problems of previous methods. In this method, like the previous methods, the telemedicine network is considered a graph network, with the difference that there are four types of nodes in this network: a real sink node, an unreal sink node, the source node, and sensor nodes. The security of the sink node includes two items: the security of the node location and the content of the node. In that article, the location of the node was given importance and the content and volume of data were not mentioned. In this method, by adding an unreal sink node and unreal data, the network becomes more complicated. The sink node produces and sends data at the same time and is not the last node in the network, thus making it difficult to identify. The problem with this method is that while the number of unreal sink nodes and the production of unreal data increases, the paths of sending information increase, and as a result, more energy and time are spent on sending data.

 One study^35^ presented a zero watermarking algorithm to prevent identity disclosure in telemedicine. The proposed algorithm embeds a person’s identity in the medical speech signals without creating any distortion. Two criteria, Hurst exponent and zero crossing, are calculated to specify the appropriate position in the signal for identity interpolation. Signal analysis shows that unvoiced speech frames are reliable in identity extraction and are also robust against noise. In the proposed zero watermarking algorithm, the identity is embedded in a secret key using a one-dimensional local binary operator instead of the signal. In one study,^36^ digital watermarking protected the privacy of patients. In that article, the proficiency of three watermarking algorithms (DWT, SVD, DWT-SVD) was considered and two different watermarking were created with the same watermarking information and different features, one of which was a QR code as a watermarking image that can carry large data in a small space while the other was a normal text image watermarking embedded in medical images. Then, the quality of the watermarking images after embedding the watermarking was obtained using two criteria (PSNR) and also subjective assessment (opinions of doctors, radiologists and medical physicists). It was found that the watermarking did not lead to loss of medical information. Experimental results show that the DWT-based methods are acceptable for medical applications where the embedding time is important, while the SVD-based methods are proper to achieve robustness.

 In another study,^38^ two targeted methods were offered against a key-based color image watermarking method as well as a non-key scheme to assess their privacy in telemedicine applications. The target methods were color image watermarking algorithms based on SVD and QR, whose embedding methods are similar. The proposed schemes use the previous knowledge of watermarking algorithms to change embedded spaces. Therefore, these changes disrupt the extraction process.According to the results, the key-based watermarking method was more secure because the proposed method for removing embedded watermarking needs to falsify the key-based watermarking images more than the non-key ones. They also perform more efficiently in watermarking removal than other common schemes such as image compression. Finally, these schemes are offered to indicate the vulnerabilities of the watermarking algorithm to help designers perform securely.

 One study^39^ proposed a privacy-preserving communication method in 5G-IoT telemedicine to gain the following features: (*i*) The process integrates telemedicine systems and emergency medical services. (*ii*) It allows emergency signals to be sent instantly by reducing the dignity of secret key leakage. (*iii*) Patient information is transmitted securely during patient transport to the destination medical institution. (*iv*) The quality of health care services is ensured by maintaining the patient’s privacy. (*v*) It supports emergency cases. (*vi*) It is resistant to possible attacks and is adequately secured using a random oracle model.

 In another research,^41^ a method was proposed to search audio recordings using fully smooth encryption on the cloud. Testing and implementation on an audio data set with various size experiments were performed while the security parameters were varied, and the proposed scheme achieved higher levels of security and privacy, especially when the security parameters were increased. However, in cases where higher levels of security are not desirable, lowering the security parameters may be done. Attribute-based signature (ABS)^42^ is an effective way of protecting users› privacy and is well suited for nameless verification and privacy control. However, these schemes involve heavy computations in the signature and acknowledgment steps, which are not suitable for devices with limited resources to access the healthcare cloud system. To solve the above problems, a lightweight medical service access and privacy scheme based on multi-authority ABS for the healthcare cloud is proposed called LPP-MSA.The proposed scheme can greatly reduce the computation costs by using online/offline signature and server-assisted verification mechanisms. In addition, LPP-MSA achieves unforgeability and anonymity. The results show that LPP-MSA is more applicable in both signing and verification phases in comparison with other existing schemes. Therefore, it can be employed where users access the healthcare cloud system for telemedicine.

 One study,^87^ focused on using cybersecurity measures based on artificial intelligence and the internet of things (IoT) to detect cyberattacks. Another study^88^ used a method for electronic health records that tries to preserve privacy using hyperledger technology and mixer identity collection. Authorized healthcare professionals can store health records and give them access to documents. Another study^89^ proposed an integrated system that preserves the privacy of various types of data related to telehealth. This medical information system merges electronic copies of electronic patient records and fingerprints, and then uses proxy and group signatures. The purpose of this study is to shift towards digitization while protecting all information. In one research,^90^ privacy issues in telemedicine using hybrid cloud systems were discussed. They applied the XACML method for accessing healthcare data stored in clouds. The main challenge in this method is the large amount of data in the cloud that needs to be securely searched without decrypting it. We summarize prominent methods for privacy preservation in telemedicine in Table 3.

**Table 3:** Comparison of Different Methods for Privacy Preservation in Telemedicine

**Method**	**Advantage**	**Disadvantage**
Blockchain based	Decentralized, tamper-proof, transparent, traceable, reliable, secure	Cost of infrastructure, lack of knowledge of implementation, lack of standardization
Graph based	Simple, secure, low cost	Time and energy consuming
Watermarking algorithm	Robustness against noise, low PSNR, preserve information	Need to be more secure
Identity based	Fast, secure, efficient for normal and emergency situation	Not mentioned
Homomorphic	High levels of security and privacy	Time-consuming
Attribute based signature	Suitable for anonymous authentication and privacy access control	Heavy computation

## Conclusion

 Telemedicine will find widespread use due to its versatility, non-invasive nature, and the daily increase in the use of the internet and communication devices. In this paper, we performed a review related to telemedicine, its application, benefits and barriers. We categorized the papers based on different diseases controlled and treated using telemedicine. Also, we reviewed the challenges in this area which showed privacy preserving of information transferred for processing through smart devices, as one of the most important challenges in the field of telemedicine. This information could be the target of cyber-attacks, which lead to the disclosure of the patients’ private information.So far, various methods have been provided to protect people’s privacy such as blockchain-based, graph-based, and identity-based methods, as well as watermarking algorithms and homomorphic and ABSs. Blockchain-based methods and graph-based methods are prominent methods in this field.
